# A Systematic Review of Cross-Cultural Adaptation and Psychometric Properties of Oral Health Literacy Tools

**DOI:** 10.3390/ijerph181910422

**Published:** 2021-10-03

**Authors:** Sobiya Praveen, Jinal Parmar, Navira Chandio, Amit Arora

**Affiliations:** 1 School of Health Sciences, Western Sydney University, Campbelltown Campus, Locked Bag 1797, Penrith, NSW 2751, Australia; drsobiyao8@gmail.com (S.P.); jinalparmar3112@gmail.com (J.P.); navira_c@yahoo.com (N.C.); 2 Health Equity Laboratory, Campbelltown, NSW 2560, Australia; 3 Translational Health Research Institute, Western Sydney University, Locked Bag 1797, Penrith, NSW 2751, Australia; 4 Clinical School Child and Adolescent Health, The Children’s Hospital at Westmead Clinical School, Faculty of Medicine and Health, The University of Sydney, Westmead, NSW 2145, Australia; 5 Oral Health Services, Sydney Local Health District and Sydney Dental Hospital, NSW Health, Surry Hills, NSW 2010, Australia

**Keywords:** oral health literacy, translation, cross-cultural adaptation, psychometric properties

## Abstract

The aims of this systematic review were to critically appraise the quality of the cross-cultural adaptation and the psychometric properties of the translated versions of oral health literacy assessment tools. CINAHL (EBSCO), Medline (EBSCO), EMBASE (Ovid), and ProQuest Dissertation and Thesis were searched systematically. Studies focusing on cross-cultural adaptation and psychometric properties of oral health literacy tools were included. The methodological quality of included studies was assessed according to the COSMIN Risk of Bias checklist. Sixteen oral health literacy instruments in 11 different languages were included in this systematic review. However, only seven instruments met the criteria for an accurate cross-cultural adaptation process, while the remaining tools failed to meet at least one criterion for suitable quality of cross-cultural adaptation process. None of the studies evaluated all the aspects of psychometric properties. Most of the studies reported internal consistency, reliability, structural validity, and construct validity. Despite adequate ratings for some reported psychometric properties, the methodological quality of studies on translated versions of oral health literacy tools was mostly doubtful to inadequate. Researchers and clinicians should follow standard guidelines for cross-cultural adaptation and assess all aspects of psychometric properties for using oral health literacy tools in cross-cultural settings.

## 1. Introduction

Oral diseases pose a significant health burden for many countries and remain to be a major global public health challenge [1]. The World Health Organization (WHO) has reported an estimated 3.5 billion people suffering from oral diseases worldwide [2]. According to the Global Burden of Disease Study 2017, oral diseases are the most common health conditions among both males and females [3]. It is estimated that between 1990 and 2015, the number of people with untreated oral diseases increased from 2.5 to 3.5 billion, causing a 64% increase in disability-adjusted life years [4]. Oral diseases affect people throughout the life course, causing pain, discomfort, sepsis, sleep loss [2], and may lead to social disruption and reduced employment potential [5]. Oral diseases disproportionally affect marginalized communities [6] and are associated with social determinants of health such as socioeconomic status, education, income, language, and health literacy [7,8,9,10].

Health literacy started as a concept associated with the ability of an individual to obtain and process information to support health actions [11]. The theoretical understandings and methods to measure health literacy have experienced a continuous evolution since its introduction in 1974 [12]. Health literacy is defined as “the degree to which individuals have the capacity to obtain, process, and understand basic health-related knowledge needed to make informed health decisions” [12]. The Calgary Charter on Health Literacy is an effort to discover core principles to support new and remodel existing health literacy framework [13], which defines health literacy as the use of a wide range of skills that allows public and health care personnel to gather, understand, communicate, and use health-related information. Therefore, health literacy is a multidimensional social construct that is not limited to reading, writing, speaking, listening, and numeracy; but also includes critical analysis, communication, and interaction skills required for healthier living [13,14]. Health literacy is a strong predictor of health behaviors and outcomes [15,16]. Limited health literacy is also associated with poor self-ratings of health, poor adherence to medical instructions, poor self-management skills, increased mortality risks, poor health outcomes, and higher healthcare costs [17,18,19,20]. The concept of oral health literacy (OHL) is similar to health literacy but is specific to dental and oral health. During the last decade, OHL has gained the attention of practitioners and policy makers due to its proven impact on oral health outcomes [21]. OHL is associated with several oral health characteristics, and those with the lowest OHL literacy had the highest risk of oral diseases [22,23,24]. OHL skills have a strong impact on an individual’s awareness of the importance of oral health, its relation to general health, and the specific health-promoting behaviors knowledge [25]. Therefore, OHL is critical in reducing oral health disparities and promoting oral health [23].

Identifying individuals with inadequate OHL is important, and several instruments have been developed to measure OHL in English speakers. The most commonly used instruments are: a) Rapid Estimate of Adult Literacy in Dentistry-30 (REALD-30) [26]; b) Rapid Estimate of Adult Literacy in Dentistry-99 (REALD-99) [27]; c) Test of Functional Health Literacy in Dentistry (ToFHLiD) [28]; and d) Oral Health Literacy Instrument (OHLI). However, these instruments lack cultural and linguistic sensitivity when applied to non-English-speaking populations [29] as it is commonly observed that some items of these instruments are not relevant to all population groups [30]. It is therefore pertinent that for using these instruments in a new country, culture, and/or language, translation, and cultural adaptation of the instrument are necessary [31,32]. In other words, the instruments require “cross-cultural adaptation”, which is a process that encompasses both language (translation) and cultural adaptation while preparing a tool for use in another setting [33]. This maintains equivalence with the original instrument and helps to ascertain if the adapted version of the instrument retains the psychometric properties [34,35].

The evidence on the psychometric properties of the instruments helps clinicians and researchers to select high-quality instruments [36]. Recently, the COnsensus-based Standards for the selection of health Measurement INstruments (COSMIN) initiative provided the criteria for evaluating the psychometric properties of instruments [37,38]. The COSMIN taxonomy includes three main domains of psychometric properties: validity, reliability, and responsiveness [39]. Since the process of cross-cultural adaptation provides only the measure of quality in content validity [33], measurement of other psychometric properties is critical to describing a successful cross-cultural adaptation.

Several reviews have been published on OHL tools [21,40,41,42,43]. In 2014, a systematic review on OHL tools concluded the need for further work required to measure OHL as a wider construct across diverse populations [40]. Recently, another systematic review examining OHL tools reported that some aspects of OHL and psychometric properties are neglected in the existing tools [41]. Other reviews [21,40,43] also focused on the dimensions measured by different OHL tools and methodology used in their development. However, to this date, no systematic review has been conducted to evaluate the quality of cross-cultural adaptation procedures and the psychometric properties of the translated version of OHL tools. Evaluation of translated versions of OHL tools is required to verify if the adapted measure retains the psychometric properties of the original instrument, as researchers have consistently reported inconsistent results for measurement properties [34,35,44]. Since it is possible that the measurement properties of OHL tools vary between nationalities, this review evaluates OHL tools according to language to reduce the inconsistencies resulting from cultural differences. Therefore, the aims of this systematic review were to synthesis evidence on the quality of translation and cross-cultural adaptation process of instruments used to assess OHL and to perform critical appraisal of the psychometric properties of translated versions of OHL tools in relation to validity, reliability, and responsiveness.

## 2. Materials and Methods

This review was reported according to the Preferred Reporting Items for Systematic Reviews and Meta-Analysis (PRISMA) guidelines [45] (Table S1). The protocol of this systematic review has been registered and published with the PROSPERO International Prospective Register of Systematic Reviews (CRD42020188812) [46].

### 2.1. Eligibility Criteria

Studies were included if one of the main aim(s) was to: translate and culturally adapt an English version of the OHL tool and evaluate the psychometric properties of translated version (s) of an OHL tool. Additionally, only studies applying quantitative study design were included. Both self-reported and objectively measured tools were included for this systematic review.

### 2.2. Information Source

The following electronic databases were searched using the specified search strategy: CINAHL (EBSCO), Medline (EBSCO), and EMBASE (Ovid). ProQuest Dissertations and Theses Global was also searched for unpublished studies. Additionally, a manual search of the reference list of all identified studies and previously published systematic reviews was also performed. No restriction was made on the publication date (i.e., from the time of inception to present), type, and region. The search was initially conducted from 9 March 2020 and then updated on 25 July 2021.

### 2.3. Search Strategy

The Population Intervention/Exposure Comparator Outcome Study design (PICOS) [47] criteria were used to devise the key concepts and related search terms. A combination of specific Medical Subject Headings (MeSH) terms and keywords related to oral or dental health literacy, tools, psychometric properties, and cross-cultural adaptation were drafted in collaboration with a professional health sciences librarian. The Boolean operators and truncation were used to narrow down and broaden the search scope. The search strategy was pre-tested in the Medline (EBSCO) database and subsequently adapted to the syntax and subject headings of the other databases (Table S2).

### 2.4. Study Selection

All the studies identified from the electronic databases, theses, and manual searches were subsequently exported to a reference manager software Endnote X9 [48] for removing duplicates, screening, and selection. Two reviewers (SP and AA) independently screened the articles based on the eligibility criteria, and manuscripts were screened by title and abstract relevance. Both the authors underwent formal training to reach a consensus for the study selection processes. Studies that were considered to potentially meet the criteria for this review were read in full text by two reviewers (SP and AA). Study authors were contacted to seek additional information in case of any uncertainty on eligibility. A total of three attempts were made to contact the study authors, and if no response was received, studies were screened for eligibility based on the information available. Disagreements were resolved through consensus with discussion with a third co-author (JP/NC/AD). The reasons for excluding the studies were reported in Table S3.

### 2.5. Data Extraction Process

Two independent reviewers (SP and AA) extracted data on the characteristics of the included OHL tools and their measurement properties (reliability, validity, and responsiveness) based on the COSMIN recommendations [39]. Data regarding translation and cross-cultural adaptation procedures of each included study were also extracted. For each instrument, data extracted included information on the publication year, country of origin, authors, type of tool, purpose, expertise of developers, development method, mode of administration, scoring categories, language, cross-cultural adaptation process, and psychometric properties. For missing data and/or uncertainties, study authors were contacted for further information with a maximum of three attempts. Where no response was received, data extraction was completed using the information available.

### 2.6. Assessment of the Methodological Quality

The methodological quality assessment of each included study was evaluated according to two checklists:

1. The Guidelines for the Process of Cross-Cultural Adaptations of Self-Report Measures [31], which states a cross-cultural adaptation, must include an initial translation, synthesis of translation, back-translation, reviews by the expert committee, and the pre-test version of the instrument. To assess the quality of the cross-cultural adaptation process, the tools were rated as + positive rating, - negative rating, 0 no information available, and unclear according to the criteria adapted from Costa and colleagues [44]. The steps of the cross-cultural adaptation process and the scoring system are described in Table S4.
2. The COnsensus-based Standards for the selection of health Measurement INstruments (COSMIN) checklist [38] was used for evaluating the psychometric properties of the translated versions of the OHL tools. This standardized checklist consists of nine boxes on measurement properties, each consisting of 3 to 38 items. Each checklist item is ranked on a 4-point scale (inadequate, doubtful, adequate, and very good). An overall score of the methodological quality of a study was determined by taking the lowest rating of any items in a box (i.e., worst-score-counts method).

Two reviewers (SP and AA) independently assessed the methodological quality of the included studies, and the disagreements were discussed with each other to reach a consensus.

### 2.7. Assessment of Psychometric Properties

Evidence for the measurement properties of included tools was extracted and assessed against the updated criteria for suitable psychometric properties [37] (Table S5). This criterion evaluates the following psychometric properties: structural validity, internal consistency, reliability, measurement error, hypothesis testing for construct validity, cross-cultural validity or measurement invariance, criterion validity, and responsiveness.

### 2.8. Data Synthesis

After data extraction, a narrative was created to provide a descriptive synthesis of the included studies in two steps. The first task was to assess the cross-cultural adaptation of all identified OHL tools. The second step was to determine the psychometric properties (reliability, validity, and responsiveness) of each tool. The quality of the cross-cultural adaptation process was analyzed on the basis of the five basic steps: initial translation, synthesis, backward translation, expert committee review, and pretesting (Table S4).

### 2.9. Measurement Properties

The measurement properties are divided into three domains: reliability, validity, and responsiveness.

#### 2.9.1. Reliability

Reliability is defined as the extent to which the results obtained are the same for repeated measurements under several conditions [39]. Reliability contains the following measurement properties:

1. *Internal consistency:* The degree of inter-relatedness among the items, expressed by Cronbach’s alpha value [39].
2. *Reliability*: The proportion of total variance in the measurements, which is because of true differences among patients, is represented by the Intraclass Correlation Coefficient (ICC) or weighted kappa [37].
3. *Measurement error:* The systematic and random error of a patient’s score that is not attributed to the true change of the construct to be measured [49]. Measurement error is calculated as the smallest detectable change or limits of agreement, and its adequacy is determined by relating them to the minimal important change [50].

#### 2.9.2. Validity

Validity is the extent to which an instrument measures the construct(s) it intends to measure. It contains the following measurement properties:

1. *Content validity:* The degree to which the content of an instrument is an adequate reflection of the construct to be measured [39]. It is assessed by asking patients and professionals about the relevance, comprehensiveness, and comprehensibility of the items, response options, and instructions [51]. Content validity is only relevant for the development of original instruments [31], and therefore, not relevant to the scope of this review.
2. *Criterion validity:* The extent to which scores of an instrument are an adequate reflection of a gold standard [39]. Since OHL tools do not have a gold standard for item selection, the domain criterion validity was not considered in this review.
3. *Construct validity:* The degree to which the scores of an instrument are consistent with hypotheses (for instance, with regard to internal relationships, relationships to scores of other instruments, or differences between relevant groups) based on the assumption that the instrument validly measures the construct to be measured [39]. It has three important aspects:

 *a.* *Structural validity:* The degree to which the scores of an instrument are an adequate reflection of the dimensionality of the construct to be measured [39]. To assess the unidimensionality of the subscale, factor analysis should be performed on each scale separately [38].
 *b.* *Hypothesis testing*: The degree to which a particular measure relates to other measures in a way one would expect if it was validly measuring the supposed construct, i.e., in accordance with predefined hypotheses about the correlation or differences between the measures [39]. The following hypothesis was set for testing the construct validity:

- Correlation with scores of instruments measuring a similar construct or another OHL tool included in the pre-specified list will be highly or moderately to highly correlated.
- Correlation with scores of instruments measuring related but not the same constructs; for example, health-related quality of life measures will be either moderately to highly or moderately correlated.
- A weak to moderate correlation will be observed between scores of instruments included here and two different subgroups of patients.

For hypothesis testing, the following predefined [52] correlation thresholds were used with overlap between the categories to allow more flexibility in the hypotheses:

- A weak correlation is defined as <0.30;
- A weak to moderate correlation is defined as >0.20 to <0.40;
- A moderate correlation is defined as >0.30 to <0.70;
- A moderate to high correlation is defined as >0.60 to <0.80;
- A high correlation is defined as >0.70.

 *c.* *Cross-cultural validity*: The degree to which the performance of items on a translated or culturally adapted instrument is an adequate reflection of the performance of items of the original version of the instrument [39]. This property is assessed by multi-group factor analysis or differential item functioning [53], using data from a population that completed the questionnaire in the original language, as well as data from a population that completed the questionnaire in the translated language.

### 2.10. Responsiveness

The ability of an instrument to detect change over time in the construct to be measured [39]. The responsiveness of the instrument is expressed as the area under the receiver operator characteristic curve [37].

## 3. Results

### 3.1. Results of the Search

**Figure 1:**
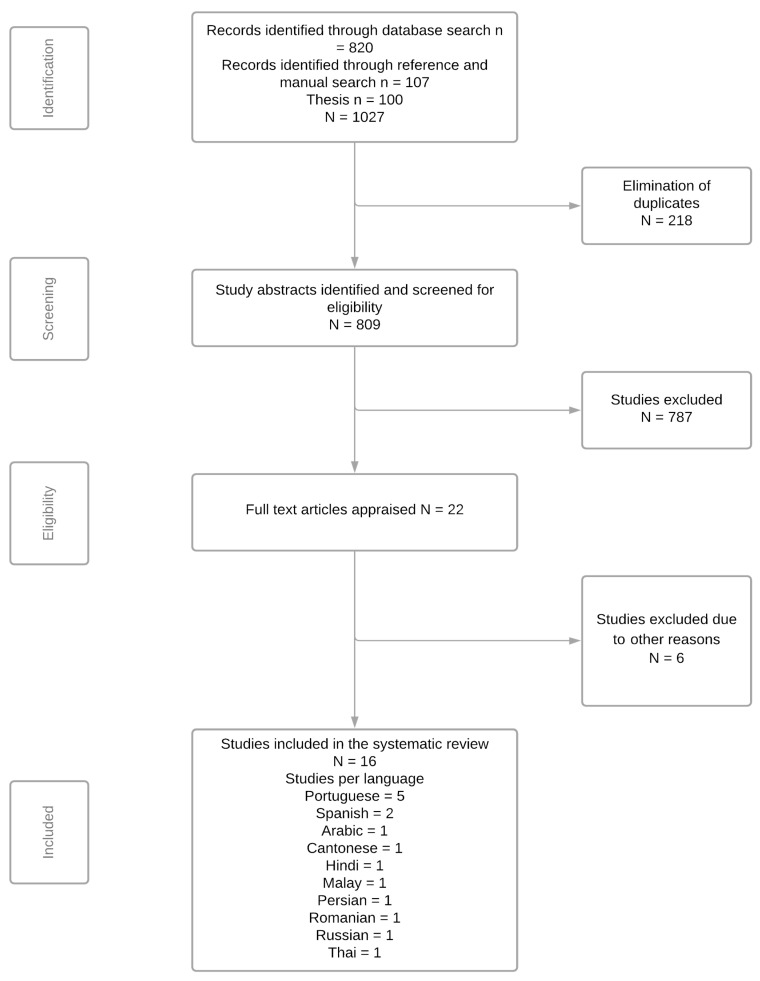
PRISMA flow diagram.

Initial searches retrieved a total of 927 articles from the electronic databases and manual search. In addition, theses and dissertations were searched, which further resulted in 100 studies. After the removal of 218 duplicates, 809 articles were identified for further reading. Of these, 786 articles were excluded as they did not measure OHL and report on the translation and cross-cultural adaptation of OHL tools. Further, one study was removed due to language limitations, and one study was removed due to accessibility issues. A total of 22 full-text studies were assessed by two authors (SP and AA), which further excluded six studies based on the eligibility criteria. The reasons for exclusion are provided in Table S3. The Cohen’s kappa value of agreement between the reviewers was 0.90, and any disagreement was resolved through a consensus and discussion process. Finally, this review included a total of 16 studies on the translated version of OHL tools evaluating instruments in 11 different languages. The identification, screening, and eligibility process are outlined in the PRISMA flow diagram (Figure 1).

### 3.2. Characteristics of Oral Health Assessment Instruments

The general overview of OHL instruments included in this review is illustrated in Table 1. All 16 instruments [54,55,56,57,58,59,60,61,62,63,64,65,66,67,68,69] were published between 2012 and 2020, indicating a recent increase in concerns regarding OHL among the non-English speaking populations. Over half of the instruments were word recognition tools [54,55,56,57,58,59,60,68,69], three were functional health literacy tools with reading comprehension and numeracy section [65,66,67], one tool was a word recognition tool with added comprehension [63], one tool encompassed comprehension, numeracy test, listening and decision-making domains [64], one tool assessed access, support, understanding, use, economic barriers, receptivity, and communication [61], and one tool assessed oral health knowledge, numeracy test and comprehension [62]. The number of items in each tool ranged from 20 to 99.

Table S6 outlines the general characteristics of translated versions of OHL tools. Most of the tools were developed by a panel of specialists with expertise in dentistry, public health, and translation in consultation with language experts. However, there were two tools [58,67] that did not report on the expertise of the developers. All the 16 tools were developed by translating the existing OHL tools, with minimal modifications to suit the needs of the identified population. Most tools were administered through face-to-face interviews conducted by the study investigators. The scoring method varied among tools; however, higher scores indicated a higher level of OHL among all tools.

The results for the cross-cultural adaptation (Table S7), psychometric properties (Table S8), and methodological quality assessment (Table S9) of different OHL instruments identified by language are presented below.

*1. Arabic*

REALD-30 is the only OHL tool that has been translated into the Arabic language [54]. REALD-30 is a word recognition test originally designed to assess the ability of an individual to read and pronounce 30 common dental words arranged in order of increasing difficulty [26]. AREALD-30 rated positive for all the steps required for an accurate translation and cross-cultural adaptation. The AREALD-30 scored sufficient (+) rating of the measurement properties for internal consistency measured by Cronbach’s α= 0.89 and test-retest reliability measured by intraclass correlation coefficient (ICC) = 0.99 (range 0.97-0.99). However, the evidence for reliability was limited due to the inadequate sample size used to perform the analysis. AREALD-30 was tested for the original one-factor structure using confirmatory factor analysis, and the results showed the presence of two factors in agreement with the original REALD-30. The unidimensionality of AREALD-30 was also evaluated by Rasch analysis, and the amount of variance explained by Rasch measures was 50.9%. A significant and positive correlation was found between AREALD-30 and AREALD-99 (Spearman’s rs = 0.95, *p* < 0.01), demonstrating very suitable convergent validity. However, the correlations of AREALD-30 with Oral Health Impact Profile (OHIP-14), self-perceived oral health status, and dental visiting habits for predictive validity were not significant. Furthermore, the discriminant validity of AREALD-30 explored across categories of the educational levels of the subjects was noted to be significant (*p* = 0.02).

*2. Chinese*

REALD-30 is the only tool translated into the traditional Chinese language [57]. HKREALD-30 rated positive for initial translation, synthesis, expert committee review, and pretesting steps. The only drawback was the lack of sufficient information about the back-translation procedure. The test-retest reliability and internal consistency of HKREALD-30 were sufficient, as shown by the ICC value of 0.78 (range 0.61–0.80, CI = 0.53–0.91) and Cronbach α value of 0.84. However, the methodological quality for reliability was inadequate as only 10% of the participants were re-interviewed after one week. Rasch analysis was used to determine the validity of the response scale and to identify redundancy using infit Z statistics. The infit ZSTD (−1.55–0.46), outfit ZSTD (−1.59–0.99), infit MNSQ (−0.84–1.07), and outfit MNSQ (0.71–1.30) for the items were within acceptable ranges. HKRERALD-30 had a highly positive and significant correlation with HKRERALD-99 (rs = 0.86, *p* < 0.01) and TOFHLiD (Table S8), reflecting adequate convergent validity. Further, there was a significant correlation (*p* < 0.01) between reading habits and HKREALD-30 (rs = 0.38 for print materials and 0.27 for digital materials). However, the correlations for other subgroups, such as their educational level and pattern of dental visits for concurrent validity, were not statistically significant.

*3. Hindi*

OHL-AQ is the only OHL tool that has been translated into the Hindi language [64]. OHL-AQ is 17 items test of functional OHL originally designed to assess four conceptual domains: reading, numeracy, listening, and decision making [70]. The forward and backward translation was performed by only one translator. The discrepancies in translation were sorted out by an expert panel, and a pretesting phase was carried out. Therefore, OHL-AQ-H did not meet the quality criteria for initial translation, synthesis, and back-translation required for the process of cross-cultural adaptation.

The internal consistency determined by Cronbach’s α value was acceptable (0.7), and the assessment of test-retest reliability among one-half of the participants after two weeks demonstrated significant results with an almost perfect agreement (ICC = 0.93, 95% CI = 0.88–0.96), indicating adequate reliability. Predictive validity and concurrent validity were reported to be significant by comparing OHL-AQ-H scores with oral hygiene status (*p* = 0.005) and dentition status (*p* = 0.001), and self-reported oral health (*p* = 0.01), respectively. However, correlation coefficients were not calculated, and no comparison with other outcome measurement instruments was performed. Therefore, the methodological quality of the OHL-AQ-H rated inadequate for construct validity.

*4. Malay*

OHLI is the only OHL tool that has been translated into the Malay language [65]. OHLI is a test of functional oral health literacy containing 38 items to assess the reading comprehension section and 19 items on numeracy skills [71]. OHLI-M rated positive for all the steps required for an accurate translation and cross-cultural adaptation process. It also scored a sufficient (+) rating for the measurement properties such as internal consistency and test-retest reliability measured by ICC = 0.86 (95% CI = 0.72–0.93). Reliability was assessed after two weeks, which makes the methodology doubtful. The Spearman’s correlation between the OHLI-M and Short Test of Functional Health Literacy in Adults was positive (rs = 0.37, *p*<0.001), supporting adequate convergent validity. However, lack of concurrent validity was indicated by the correlations between OHLI-M scores and decayed, missing, and filled teeth (DMFT) index (Pearson’s correlation r = −0.11, *p* = 0.33) and Community Periodontal Index (CPI) scores (r = −0.04, *p* = 0.70). The OHLI-M scores among categories of education (*p*<0.001) and time since the last dental visit (*p*=<0.020) were significant.

*5. Persian*

REALD-99 is the only OHL tool that has been translated into the Persian language [58]. REALD-99 is a word recognition test originally made up of 99 common dental words with varying levels of difficulty [27]. IREALD-99 rated positive for the quality criteria for initial translation, synthesis, back-translation, and pre-test. However, no information about the existence of an expert committee was provided. The project manager compared the translation versions and reconciled discrepancies before pretesting.

Internal consistency was higher than 0.70, and the test-retest reliability was also sufficient (Table S8) on administration after two weeks. A principal component analysis was performed to assess unidimensionality and strong first factor. The variance explained by Rasch measures was 47.54%. The methodological quality for structural validity was considered inadequate due to the sample size. The convergent validity of IREALD-99 was supported by the positive correlation between TOFHLiD scores and self-perceived dental health status as outlined in Table S8. For concurrent validity, IREALD-99 was compared across education and income categories. There were significant differences (*p* < 0.01) in the IREALD-99 scores across educational categories, but not income levels (*p* = 0.09).

*6. Portuguese*

There are five OHL instruments that have been translated into the Brazilian-Portuguese language: REALD-30 [55], REALMD-20 [56], OHLA-S [63], HKOHLAT-P [62], and HeLD [61]. REALMD-20 is a singular tool containing 20 items designed to screen patients by their ability to read medical and dental words [72]. OHLA-S is a pronunciation and comprehension test originally containing 30 items related to the oral conditions with an added comprehension test for the use in Spanish speakers [73]. HKOHLAT-P is a tool originally developed to be used in Hong Kong [74]. It evaluates oral health knowledge, reading comprehension, and numeracy and is mainly focused on pediatric dentistry [62]. HeLD is a tool originally containing 29 items for assessing multiple dimensions for OHL encompassing communication, access, receptivity, understanding, use, support, and economic barriers [75].

*Brazilian-HeLD*: Brazilian-HeLD rated positive for all the steps required for an accurate translation and cross-cultural adaptation processes, except for the synthesis after the initial translation. HeLD scale comprises HeLD-29 and HeLD-14. All the seven factors in both forms of HeLD had adequate internal consistency (Table S8). However, the evidence for reliability was unknown as the ICC value was not reported. Confirmatory factor analysis was performed to test the fit of data to the factor structure of both HeLD forms. However, the goodness of fit of the confirmatory factor analysis models demonstrated satisfactory results only for HeLD-14 subsamples (CFI = 0.97–0.98; RMSEA = 0.05 and SRMR = 0.03). Convergent validity was estimated by calculating the average variance extracted and composite reliability. However, no comparison with other outcome measurement instruments was performed, and correlation coefficients were not reported.

*BOHLAT-P:* BOHLAT-P rated positive for all the steps required for an accurate translation and cross-cultural adaptation process. The reliability of the BOHLAT-P evaluated through the assessment of internal consistency, and test-retest (Table S8) were well above the recommended levels. Exploratory factor analysis was performed to evaluate the dimensionality of BOHLAT-P, following which confirmatory factor analysis was performed to confirm the unidimensionality. The goodness-of-fit indices was X2 = 1506.530, df = 1124, CFI = 0.934, TLI = 0.931, and RMSEA = 0.041, thus indicating an acceptable to excellent model fit. The only limitation in determining structural validity was the inadequate sample size. Convergent validity of BOHLAT-P measured by Spearman’s correlation test showed a high positive statistically significant correlation with BREALD-30 scores, the number of years of schooling, and the number of hours spent reading as outlined in Table S8. BOHLAT-P scores had a negative correlation with Early Childhood Oral Health Impact Scale scores and the number of cavitated teeth. After controlling for confounding variables, the associations of BOHLAT-P scores with caries or the number of teeth with cavitated dental caries (tooth decay) were not significant.

*BREALD-30:* BREALD-30 rated positive for all the steps required for an accurate translation and cross-cultural adaptation process. It demonstrated a suitable internal consistency, scored excellent for test-retest reliability, and had moderate to nearly perfect kappa coefficients ranging from 0.42 to 1.00. However, an inadequate sample size was a limitation. Exploratory factor analysis was performed to assess the unidimensionality of the instrument, and the result demonstrated the predominance of one factor. Similar to the original study, the hypothesis that OHL measured by the BREALD-30 is unidimensional was not confirmed, and at least seven factors were necessary to explain 50% of the total variance. However, no confirmatory factor analysis was performed, due to which the structural validity was indeterminate. Convergent validity accessed by correlating the BREALD-30 and scores with the level of general literacy measured by the National Functional Literacy Index and educational attainment was statistically significant (Table S8). The test for discriminant validity showed statistically significant differences according to the occupation (*p* = 0.004), a history of dental visits (*p* = 0.017) and monthly household income (*p* < 0.001). No significant correlation was found between the BREALD-30 and OHIP-14 scores (rs = −0.08; *p* = 0.198). The BREALD-30 score was significantly associated with the respondent’s assessment of his/her child’s oral health after adjusting for other covariates (*p* = 0.024).

*BREALMD-20:  *BREALMD-20 rated positive for initial translation, synthesis, back-translation, and pretesting steps required for an accurate translation and cross-cultural adaptation process. However, there was no information regarding the existence of an expert committee to verify the translated versions. The internal consistency was above the recommended level. Although the test-retest reliability assessed after one month was also considered sufficient (ICC = 0.73, 95% CI = 0.66–0.79), the methodological quality was lowered due to the inadequate sample size. The health literacy measured by REALMD-20 was found to be multidimensional. The first four factors accounted for 52.1% of the total variance. No confirmatory factor analysis was performed, due to which the rating for structural validity was indeterminate. A positive and significant correlation was found between the REALMD-20 and the BREALD-30 (rs = 0.73, *p* <0.001) and Brazilian National Functional Literacy Index (rs = 0.60, *p* < 0:001), reflecting very suitable convergent validity. When compared for discriminant validity across categories, BREALMD-20 scores were higher among health professionals, more educated people, individuals who reported good/excellent oral health conditions, and who sought preventive dental services.

*OHLA-B:* OHLA-B rated positive for all the steps required for an accurate translation and cross-cultural adaptation process. The psychometric analysis of OLHA-B was not performed.

*7. Romanian*

REALD-30 is the only tool that has been translated into the Romanian language [60]. RREALD-30 rated positive for forward translation, synthesis, and pretesting steps. However, back-translation into English was performed by a single translator, and although the existing committee agreed on the first Romanian version, the design was doubtful. The internal consistency and the test-retest reliability were adequate. However, the evidence for reliability was limited due to the small sample size used for the analysis.

RREALD-30 was tested for the original one-factor structure using principal component analysis, and the results showed RREALD-30 had a one-factor solution. The unidimensionality evaluated by Rasch model analysis demonstrated a discriminating ability for each RREALD-30 word. The sum score of RREALD-30 applied in a structural equation model showed an adequate fit (Table S8). RREALD-30 demonstrated suitable concurrent and predictive validity. A significant correlation (*p* < 0.001) of RREALD-30 scores was found with sex, education, and dental visits. The RREALD-30 scores had a statistically significant effect also on OHIP-14 (*p* = 0.004). An important drawback was the lack of comparison with other validated health literacy tools required to assess the convergent validity.

*8. Russian*

OHLI is the only tool that has been translated into the Russian language [67]. In contrast to all other translated versions, it failed to meet the criteria for a positive rating for any of the steps involved in the translation process. R-OHLI was translated from English to Russian by only one translator, followed by back-translation made by an independent translator. Two translators then evaluated the equivalence between the original and back-translated versions. The translated versions did not do through review by an expert committee, which is a critical step in finalizing the prefinal and final versions. Moreover, the prefinal version was not tested in a sample of the population.

R-OHLI scored a sufficient (+) rating for the measurement properties such as internal consistency and test-retest reliability (Table S8). Despite this, the evidence for reliability was low because of the very small sample size used for the analysis. For construct validity, R-OHLI was compared with the oral health knowledge test, which showed a significant correlation (rs = 0.363, *p*< 0.001). A significant drawback was the lack of comparison with similar outcome measure instruments and the use of the non-validated tool for comparison. Therefore, methodological quality for construct validity was inadequate.

*9. Spanish*

OHLI [66] and REALD-30 [59] have been translated into the Spanish language. OHLI-Cl: OHLI-Cl rated positive for all the steps required for an accurate translation and cross-cultural adaptation. For OHLI-CI, the internal consistency was high, and reliability was sufficient, as outlined in Table S8. However, the methodological quality for reliability was doubtful due to the inadequate sample size.

The Pearson and Spearman correlations to determine the convergent validity of OHLI-Cl for Oral Health Knowledge Test and for Short Assessment of Health Literacy for Spanish-speaking Adults (SAHLSA) were statistically significant. For predictive validity, the correlations of the OHLI-Cl with DMFT, CPI, Oral Hygiene Index Simplified (OHIS), and Oral Health Impact Profile of 49 items (OHIP-49) were determined, which were also statistically significant (*p* < 0.01). A significant disadvantage was the lack of comparison with a validated instrument measuring similar outcome measures, and hence the evidence for construct validity was inadequate.

*REALD-30 for the Chilean population:* The forward translation of the instrument by two independent native Spanish speakers met the criteria of an accurate translation process, followed by synthesis to produce a consensus. However, back-translation was not reported, and pretesting was not performed. Although an evaluation was made by four experts in dental public health, the design is doubtful. The internal consistency was high, and the reliability was sufficient in a retest performed after four weeks (Table S8). Despite that, the methodological quality for reliability was doubtful due to the inadequate sample size. For predictive and convergent validity, Pearson’s r and Spearman’s rho correlation coefficients were estimated. A significant strong positive association of Spanish REALD-30 was found with SAHLSA (r = 0.71; rs = 0.69 <0.01). The correlation with CPI, OHIS, DMFT, and OHIP-49sp was also statistically significant.

*10. Thai*

REALD-30 is the only tool that has been translated into the Thai language [68]. ThREALD-30 rated positive for the initial translation, synthesis, and pre-test steps of the cross-cultural adaptation process. The authors reported that the back-translation and expert committee review were performed; however, there was no information on the number of translators involved, and the review process was also doubtful.

The internal consistency and pre-posttest reliability of ThREALD-30 were excellent (Table S8). Even so, evidence for reliability is limited due to doubtful methodological quality. ThREALD-30 had a significant negative correlation with OHIP-14 Spearman’s rank correlation coefficient, oral health status, DMFT, OHIS, and clinical attachment loss, respectively (Table S8). A significant drawback was the lack of comparison with other validated instruments to measure OHL, due to which the methodological quality for construct validity was inadequate.

*11. Turkish*

REALD-30 is the only tool that has been translated into the Turkish language [69] and rated positive for all the steps required for an accurate translation and cross-cultural adaptation.

The internal consistency and the test-retest reliability are well above the recommended levels (Table S8). Classical Test Theory and Rasch analysis were performed, and both suggested OHL as multidimensional. The results of CFA indicated that the two-factor model demonstrated a better fit than did the one-factor model (x^2^/df=1.34, CFI=0.89, TLI=0.89, and RMSEA=0.052). The Rasch analysis explained 37.9% of the total variance in this data set. However, the sample size used in the analysis was less than five times the number of items; hence the methodological quality was inadequate. TREALD-30 was positively and significantly associated with REALM, as well as with the participants’ reading ability of hospital materials, thus indicating its convergent validity. TREALD-30 scores were weak but significantly correlated with the number of missing teeth, age, OHIP-14 score, years of schooling, self-rated oral health, and family monthly income. Further, there was a significant association between the use of dental floss and daily consumption of sugar-added food and beverages, suggesting the predictive validity of TREALD-30.

## 4. Discussion

The results showed that of the 15 studies that performed psychometric analysis, most studies examined internal consistency [54,55,56,57,58,59,60,61,62,64,65,66,67,68,69], reliability [54,55,56,57,58,59,60,62,64,65,66,67,68,69], construct validity [54,55,56,57,58,59,60,61,62,64,65,66,67,68,69], and structural validity [54,55,56,57,58,60,61,62,69]. None of the studies reported on the cross-cultural validity, measurement error, and responsiveness.

For the selection of instruments in different languages and cultures, WHO recommends the translation and cross-cultural adaptation of existing instruments [76], thereby improving communication between patients and healthcare providers and drawing international comparisons. All tools were included in the review [54,55,56,57,58,59,60,61,62,63,64,65,66,67,68,69]; however, only seven tools followed all the steps required for an accurate translation process [54,55,62,63,65,66,69]. The main reason behind poor ratings was the lack of detailed information provided for the cross-cultural adaptation process. A poor translation process creates inconsistencies between the translated and original versions of instruments, which can affect the validity of the instrument [77]. The results of this review indicate that the process of translation did not affect the reliability of an instrument. Despite poor translation and cross-cultural adaptation processes, most tools had high reliability and internal consistency reflected by the ICC and Cronbach’s α values, respectively. Generally, the methodological quality of the translation process rated negative, mainly due to the involvement of a single translator both in the forward and backward translations. It is recommended that the translation processes should be performed by at least two independent translators to ensure the translated version reflects the same item content as the original version [78]. Moreover, many studies did not report on the clear existence of the expert committee and, if reported, were of doubtful design. It is recommended that an expert committee comprising of methodologists, health professionals, language professionals, and translators should reach a consensus on any discrepancy to develop a prefinal version for field testing, which is crucial to achieving cross-cultural equivalence [79].

The COSMIN checklist provides a separate determination of the methodological quality of the studies and their results to compare the psychometric properties of instruments during a systematic review. This approach provides an independent quality rating scores for each psychometric property, making it advantageous over other tools [80]. The most reported psychometric property was internal consistency expressed by Cronbach α, and the results were adequate [54,55,56,57,58,59,60,61,62,64,65,66,67,68,69]. Furthermore, the methodological quality of the studies for internal consistency was also generally very good. Internal consistency ascertains the uniformity of the measures [81]. Higher values represent a strong correlation between the items of a scale, and values higher than 0.9 indicate that some items are not essential and can be omitted to shorten the scale [82].

The ICC values for reliability were adequate for most studies (≥0.70) [54,55,56,57,58,59,60,62,64,65,66,67,68,69]. However, the methodological quality was mostly doubtful or inadequate due to lack of evidence provided for similar test conditions, inappropriate time interval, and inadequate sample size used to perform analysis [54,55,56,57,58,59,60,65,66,67,68]. ICC analyses test-retest, interrater, and interrater reliability reflect both the extent of correlation and agreement between measurements [83]. It is, therefore, necessary to assess the ICC reliability of an instrument before its application in research or clinical use.

Among the nine studies that reported on structural validity [54,55,56,57,58,60,61,62,69], six had very good or adequate methodological quality [54,55,56,57,60,61]. The inadequate sample size was the common methodological shortcoming of the remaining studies [58,62,69]. Structural validity assesses the dimensional structure of an instrument through factor analyses [84]. The purpose of factor analysis is to provide data on variables and items of a questionnaire that can be reduced to facilitate the understanding of underlying concepts and their interpretation [85].

Although most of the studies reported statistically significant associations, there were only 10 studies that had adequate or very good methodological quality [54,55,56,57,58,59,62,65,66,69]. The common reasons for inadequate methodological quality for construct validity were lack of comparison with validated health literacy tools used in a similar setting, correlation coefficients not calculated, and inappropriate sample size. A correct hypothesis for the construct validity of an instrument provides evidence that the instrument measures what it is intended to measure [52].

From the results, it was found that there is no comprehensive OHL tool to examine all the domains and psychometric properties according to the COSMIN checklist. Despite high values for the reported psychometric properties, it is important to note that the quality of the majority of the studies was recorded as doubtful or inadequate. Moreover, none of the studies evaluated measurement error, cross-cultural validity, and responsiveness, which is very concerning. It is important to know the minimal important change in scores of OHL to understand whether the smallest measured changes in literacy level are meaningful and matter to the patients [86]. Information regarding the cross-cultural validity of the tools using measurement variance analysis is also required to know differences between group factors such as age, sex, or different patient populations. It is possible that differences between groups allow them to respond differently to a particular item [38]. Similarly, the responsiveness of an instrument is important to measure the changes in measurements over time [87]. However, due to the cross-sectional nature of the included studies, the ability of OHL tools to measure OHL over time was not reported.

The existing OHL tools measure word recognition, numeracy, and reading skills related to oral health context [42]. The wider range of skills required for decision making, communication, and health care use must be incorporated into the tools to capture all dimensions of OHL. Low health literacy, limited English proficiency, and cultural barriers are identified as a “triple threat” to effective communication between patients and healthcare providers [88]. Health literacy is an emerging field, and integration of cultural and linguistic is necessary to provide competent care. The original versions of tools are less useful to assess OHL levels in non-English speaking countries.

### 4.1. Strengths and Limitations

To the best of our knowledge, this is the first systematic review to evaluate the cross-cultural adaptation and psychometric properties of OHL tools in languages other than English. This systematic review followed standard guidelines for the process of translation and cross-cultural adaptation and the COSMIN checklist in reporting psychometric analysis of the OHL tools, which provides a standardized, detailed, and transparent framework to evaluate measurement properties of health outcome measurement instruments. The COSMIN guidelines were preferred over other methods due to the advantage of assessing the quality of all domains of psychometric properties comprehensively, while other methods were designed for evaluating only limited aspects of psychometric properties such as criterion validity [89] or reliability [90]. Another strength is the review evaluated translated versions of OHL tools by language, which facilitates a selection of the best tool available in that language.

This systematic review has a few limitations. First, we only included studies published in the English language, and it is, therefore, possible that we might have missed some translated versions of OHL tools published in non-English journals. Second, one study was excluded due to inaccessibility despite repeated attempts to contact the authors, which could have provided further insights. Third, we used the “the worst score counts” principle, as per the COSMIN Risk of Bias checklist, which means the methodological quality is interpreted by taking the lowest score achieved for a psychometric property and that poor aspects of the study cannot be compensated by the suitable aspects of the study. For example, even if one of the several items in an instrument scores inadequate, then an overall rating of that psychometric property is reported as inadequate. Finally, only three databases were searched, so it is possible that some relevant studies may have been missed.

### 4.2. Implications

The findings of this review may provide important information to the relevant stakeholders, including oral and dental health professionals, treatment teams, and researchers, regarding the approaches to measure OHL in a culturally and linguistically diverse population. Since there is a lack of a substantial amount of information on psychometric properties and poor assessment or reporting of the studies, it is very challenging to recommend which tool is the best. Therefore, high-quality studies are required to fill gaps in knowledge regarding different aspects of cross-cultural adaptation and psychometric analysis of OHL tools. There is a need for accurately cross-culturally adapted tools with suitable reliability, validity, and responsiveness to measure OHL in non-English speaking countries where the prevalence of diseases is disproportionally higher.

## 5. Conclusions

The quality of translations and cross-cultural adaptation was poor, and none of the tools were evaluated for all the aspects of psychometric properties. A significant amount of information regarding the cross-cultural adaptation process and psychometric properties was missing or had doubtful methodological quality. There is no comprehensive tool that evaluates all aspects of psychometric properties in cross-cultural settings. Despite promising values for some measurement properties, the evidence for reliability and validity is limited due to methodological deficiencies. Future studies on cross-cultural adaptation should emphasize the use of multiple bilingual translators and expert panel roles in the process. Further work is required to develop tools by incorporating all aspects of psychometric analysis to ensure clinical utility and cultural competence.

## Figures and Tables

**Table 1:** Overview of OHL instruments.

Abbreviation	Name of Instrument	Year	Country	Authors	Type of Tool
AREALD-30 [54]	Arabic Rapid Estimate of Adult Literacy in Dentistry	2014	Saudi Arabia	Tadakamadla et al.	30-item word recognition tool
Brazilian-HeLD [61]	Brazilian-Portuguese version of Health Literacy in Dentistry (HeLD) scale	2020	Brazil	Mialhe et al.	29-item (HeLD-29) and 14-item (HeLD-14) tools assessing access, support, understanding, use, economic barriers, receptivity, and communication
BOHLAT-P [62]	Brazilian-Portuguese version of the Hong Kong OHL Assessment Task for Pediatric Dentistry (HKOHLAT-P)	2020	Brazil	Firmino et al.	49-item tool assessing oral health knowledge, numeracy test and comprehensive
BREALD-30 [55]	Brazilian version of the Rapid Estimate of Adult Literacy in Dentistry	2015	Brazil	Junkes et al.	30-item word recognition tool
BREALMD-20 [56]	Brazilian version of 20-item Rapid Estimate Adult Literacy in Medicine and Dentistry	2017	Brazi	Cruvinel et al.	20-item word recognition tool
HKREALD-30 [57]	Hong Kong Rapid Estimate of Adult Literacy in Dentistry	2012	Hong Kong	Wong et al.	30-item word recognition tool
IREALD-99 [58]	Rapid Estimate of Adult Literacy in Dentistry-99 for Iranian population	2016	Iran	Pakpour et al.	99-item word recognition tool
OHLA-B [63]	Oral Health Literacy Assessment—Brazilian	2017	Brazil	Bado et al.	30-item word recognition and comprehension tool
OHL-AQ-H [64]	Oral Health Literacy Adult Questionnaire—Hindi Version	2016	India	Vyas et al.	17-item tool encompassing comprehension, numeracy test, listening, and decision-making domains
OHLI-Cl [66]	Chilean version of OHLI	2017	Chile	Cartes-Velásquez and Luengo-Machucaa	57-item functional health literacy tool (38 items for reading comprehension and 19 items for numeracy test)
OHLI-M [65]	Oral health literacy instrument—Malay version	2020	Malaysia	Ramlay et al.	57-item functional health literacy tool (38 items for reading comprehension and 19 items for numeracy test)
REALD-30 for Chilean population [59]	Rapid Estimate of Adult Literacy in Dentistry for Chilean population	2018	Chile	Cartes- Velásquez and Luengo-Machucaa	30-item word recognition tool
R-OHLI [67]	Russian version of the OHLI	2014	Belarus	Blizniuk et al.	57-item functional health literacy tool containing 38 items for reading comprehension and 19 items for a numeracy test
RREALD-30 [60]	Romanian rapid estimate of adult literacy in dentistry	2020	Romania	Sfeatcu et al.	30-item word recognition tool
ThREALD-30 [68]	Thai version of Rapid Estimate of Adult Literacy in Dentistry	2019	Thailand	Deeraksa et al.	30-item word recognition tool
TREALD-30 [69]	Turkish version of Rapid Estimate of Adult Literacy in Dentistry-30	2017	Turkey	Peker et al.	30-item word recognition tool

OHL–Oral Health Literacy.

## Data Availability

The data presented in this study are not publicly available due to privacy.

## Supplementary Materials

The following are available online at https://www.mdpi.com/article/10.3390/ijerph181910422/s1, Table S1: PRISMA Checklist, Table S2: MEDLINE (EBSCO) search strategy, Table S3: Reasons for excluded studies, Table S4: Guidelines for the process of the cross-cultural adaptation of self-reported measures (adapted from Costa et al.), Table S5: Updated criteria for suitable measurement properties, Table S6: Characteristics of oral health assessment instruments, Table S7: Assessment of quality of translation and cross-cultural adaptation of oral health literacy tools into different languages, Table S8: Rating of the measurement properties per language and tool using the criteria for the suitable psychometric properties and Table S9: Methodological Quality Assessment of Studies on Psychometric Properties of the Included Tools using COSMIN risk of bias checklist.

## Abbreviations

AREALD-30	Arabic Rapid Estimate of Adult Literacy in Dentistry
Brazilian-HeLD	Brazilian-Portuguese version of Health Literacy in Dentistry scale
BOHLAT-P	Brazilian-Portuguese version of the Hong Kong OHL Assessment Task for Pediatric Dentistry
BREALD-30	Brazilian version of the Rapid Estimate of Adult Literacy in Dentistry
BREALMD-20	Brazilian version of 20-item Rapid Estimate Adult Literacy in Medicine and Dentistry
COSMIN	COnsensus-based Standards for the selection of health Measurement Instruments
CPI	Community Periodontal Index
DMFT	Decayed, Missing, and Filled Teeth
HKREALD-30	Hong Kong Rapid Estimate of Adult Literacy in Dentistry
ICC	Intraclass Correlation Coefficient
IREALD-99	Rapid Estimate of Adult Literacy in Dentistry-99 for Iranian population
MeSH	Medical Subject Headings
OHIP	Oral Health Impact Profile
OHIS	Oral Hygiene Index Simplified
OHL	Oral Health Literacy
OHLA-B	Oral Health Literacy Assessment-Brazilian
OHL-AQ-H	Oral Health Literacy Adult Questionnaire-Hindi Version
OHLI	Oral Health Literacy Instrument
OHLI-Cl	Chilean version of OHLI
OHLI-M	Oral health literacy instrument –Malay version
PICOS	the Population, Intervention, Comparator, Outcome, and Study Design
PRISMA	Preferred Reporting Items for Systematic Reviews and Meta-Analysis
PROSPERO	International Prospective Register of Systematic Reviews
REALD-30	Rapid Estimate of Adult Literacy in Dentistry
R-OHLI	Russian version of Oral Health Literacy Instrument
RREALD-30	Romanian version of Rapid Estimate of Adult Literacy in Dentistry-30
SAHLSA	Short Assessment of Health Literacy for Spanish-speaking Adults
ToFHLiD	Test of Functional Health Literacy in Dentistry
ThREALD-30	Thai version of Rapid Estimate of Adult Literacy in Dentistry-30
TREALD-30	Turkish version of Rapid Estimate of Adult Literacy in Dentistry-30
WHO	World Health Organization
