# Supplementary material for: A Systematic Review of Cross-Cultural Adaptation and Psychometric Properties of Oral Health Literacy Tools
Source: Int J Environ Res Public Health. 2021 Oct 3;18(19):10422. doi: 10.3390/ijerph181910422 (PMC8508111; doi:10.3390/ijerph181910422)
Supplement: Supplementary file 1 [file ijerph-18-10422-s001.zip › ijerph-1338437-supplementary.pdf]

**Supplementary Material**

**Table S1**. PRISMA Checklist

| Section/topic | # | Checklist item | Reported on page # |
|---|---|---|---|
| **TITLE** | | | |
| Title | 1 | Identify the report as a systematic review, meta-analysis, or both. | 1 |
| **ABSTRACT** | | | |
| Structured summary | 2 | Provide a structured summary including, as applicable: background; objectives; data sources; study eligibility criteria, participants, and interventions; study appraisal and synthesis methods; results; limitations; conclusions and implications of key findings; systematic review registration number. | 1 |
| **INTRODUCTION** | | | |
| Rationale | 3 | Describe the rationale for the review in the context of what is already known. | 1-3 |
| Objectives | 4 | Provide an explicit statement of questions being addressed with reference to participants, interventions, comparisons, outcomes, and study design (PICOS). | 3 |
| **METHODS** | | | |
| Protocol and registration | 5 | Indicate if a review protocol exists, if and where it can be accessed (e.g., Web address), and, if available, provide registration information including registration number. | 3 |
| Eligibility criteria | 6 | Specify study characteristics (e.g., PICOS, length of follow-up) and report characteristics (e.g., years considered, language, publication status) used as criteria for eligibility, giving rationale. | 3 |
| Information sources | 7 | Describe all information sources (e.g., databases with dates of coverage, contact with study authors to identify additional studies) in the search and date last searched. | 3 |
| Search | 8 | Present full electronic search strategy for at least one database, including any limits used, such that it could be repeated. | 3 |
| Study selection | 9 | State the process for selecting studies (i.e., screening, eligibility, included in systematic review, and, if applicable, included in the meta-analysis). | 3-4 |

| | | | |
|---|---|---|---|
| Data collection process | 10 | Describe method of data extraction from reports (e.g., piloted forms, independently, in duplicate) and any processes for obtaining and confirming data from investigators. | 4 |
| Data items | 11 | List and define all variables for which data were sought (e.g., PICOS, funding sources) and any assumptions and simplifications made. | 3-4 |
| Risk of bias in individual studies | 12 | Describe methods used for assessing risk of bias of individual studies (including specification of whether this was done at the study or outcome level), and how this information is to be used in any data synthesis. | 4-5 |
| Summary measures | 13 | State the principal summary measures (e.g., risk ratio, difference in means). | N/A |
| Synthesis of results | 14 | Describe the methods of handling data and combining results of studies, if done, including measures of consistency (e.g., $I^2$) for each meta-analysis. | 5-6 |
| Risk of bias across studies | 15 | Specify any assessment of risk of bias that may affect the cumulative evidence (e.g., publication bias, selective reporting within studies). | 4-5 |
| Additional analyses | 16 | Describe methods of additional analyses (e.g., sensitivity or subgroup analyses, meta-regression), if done, indicating which were pre-specified. | N/A |
| **RESULTS** | | | |
| Study selection | 17 | Give numbers of studies screened, assessed for eligibility, and included in the review, with reasons for exclusions at each stage, ideally with a flow diagram. | 6-7 |
| Study characteristics | 18 | For each study, present characteristics for which data were extracted (e.g., study size, PICOS, follow-up period) and provide the citations. | 10 |
| Risk of bias within studies | 19 | Present data on risk of bias of each study and, if available, any outcome level assessment (see item 12). | 10-15 |
| Results of individual studies | 20 | For all outcomes considered (benefits or harms), present, for each study: (a) simple summary data for each intervention group (b) effect estimates and confidence intervals, ideally with a forest plot. | 10-15 |
| Synthesis of results | 21 | Present results of each meta-analysis done, including confidence intervals and measures of consistency. | 10-15 |
| Risk of bias across studies | 22 | Present results of any assessment of risk of bias across studies (see Item 15). | 10-15 |
| Additional analysis | 23 | Give results of additional analyses, if done (e.g., sensitivity or subgroup analyses, meta-regression [see Item 16]). | N/A |

| | | | |
|---|---|---|---|
| **DISCUSSION** | | | |
| Summary of evidence | 24 | Summarize the main findings including the strength of evidence for each main outcome; consider their relevance to key groups (e.g., healthcare providers, users, and policy makers). | 15-18 |
| Limitations | 25 | Discuss limitations at study and outcome level (e.g., risk of bias), and at review-level (e.g., incomplete retrieval of identified research, reporting bias). | 17 |
| Conclusions | 26 | Provide a general interpretation of the results in the context of other evidence, and implications for future research. | 17-18 |
| **FUNDING** | | | |
| Funding | 27 | Describe sources of funding for the systematic review and other support (e.g., supply of data); role of funders for the systematic review. | 18 |

*From:* Moher D, Liberati A, Tetzlaff J, Altman DG, The PRISMA Group (2009). Preferred Reporting Items for Systematic Reviews and Meta-Analyses: The PRISMA Statement. PLoS Med 6(7): e1000097. doi:10.1371/journal.pmed1000097

For more information, visit: www.prisma-statement.org.

**Table S2.** MEDLINE (EBSCO) search strategy

| S.N | MeSH Terms | Output |
|---|---|---|
| 1. | (MM "Oral Health") OR "Oral health" | 43,797 |
| 2. | (MH "Dentistry+") | 104,672 |
| 3. | "Dental health" | 10,774 |
| 4. | "Oral" | 802,501 |
| 5. | "Dental" | 406,308 |
| 6. | S1 OR S2 OR S3 OR S4 OR S5 | 1,145,959 |
| 7. | (MH "Health Literacy") OR "Health literacy" | 29,216 |
| 8. | (MH "Health") OR "health" | 6,620,164 |
| 9. | (MH "Literacy" OR "literacy" | 131,720 |
| 10. | S8 AND S9 | 45,225 |
| 11. | S7 OR S8 OR S9 OR S10 | 6,706,659 |
| 12. | S6 AND S11 | 192,728 |
| 13. | (MH "Surveys and Questionnaires") OR "Questionnaire" OR "survey" | 2,165,368 |
| 14. | "Tool*" | 1,671,555 |
| 15. | "Instrument*" | 1,017,349 |
| 16. | "Index" | 1,532,768 |
| 17. | S13 OR S14 OR S15 OR S16 | 5,733,688 |
| 18. | (MH "Psychometrics") OR "Psychometric" | 176,196 |
| 19. | (MH "Reproducibility of Results") OR (MH "Validation Study") OR "Validity" | 625,209 |
| 20. | "Reliability" | 556,294 |
| 21. | S18 OR S19 OR S20 | 1,055,393 |
| 22. | "adapt*" OR "translat* or validat*" | 1,270,349 |
| 23. | S12 AND S17 AND S21 AND S22 | 416 |

**Table S3.** Reasons for excluded studies

| Author(s) and Year | Reasons for Exclusion |
|---|---|
| (Pereira Cruvinel et al., 2018) | Instrument used for screening general health literacy |
| (Wanichsaithong, 2019) | Focused on the development of new oral health literacy tool for older adults |
| Taoufik et al., 2020 | Focused on development of new oral health literacy instrument in Greek |
| Sermsuti-Anuwat, N., & Pongpanich, S. (2019) | Used among Thai adults with physical disabilities |
| Lee et al., 2013 | Not a translated version of already validated oral health literacy tool |
| Wong et al., 2013 | Focussed on development of new oral health literacy tool in Chinese |

**Table S4.** Guidelines for the process of the cross-cultural adaption of self-reported measures (adapted from Costa et al.) [44]

| Steps | Description | Rating scheme |
|---|---|---|
| Translation | Two (or more) translators should independently translate the original questionnaire. The translators should preferably be native speakers to target language. | + Translation performed by at least two independent translators<br>? Doubtful translation procedure<br>- Translation performed by only one translator<br>0 No information about translation |
| Synthesis | The translators should synthesize the multiple translations to produce a consensus of the translations. | + Performed synthesis<br>? Doubtful design<br>0 No information about synthesis OR translation performed by only one translator. |
| Back Translation | Translators, blinded to the original questionnaire shoulder translate the consensus translation back into the original language. | + Back translation performed by at least two independent translators<br>? Doubtful back translation procedure<br>- Back translation performed by only one translator<br>0 No information about back translation |
| Expert Committee Review | The expert committee should consolidate all the versions of the questionnaire and develop what would be considered the prefinal version of the questionnaire for testing. | + Clearly reported the existence of an expert committee<br>? Doubtful design<br>0 No information about the expert committee |

| | | |
|---|---|---|
| Pretesting | The prefinal questionnaire undergoes pilot testing with members of the target population. | + Performed pretesting<br>? Doubtful design<br>0 No information |

+= positive rating; - = negative rating; 0= no information available; ?=unclear

**Table S5.** Updated criteria for good measurement properties [37]

| **Measurement Property** | **Rating** | **Criteria** |
|---|---|---|
| **Structural validity** | + | **CTT:**<br>CFA: CFI or TLI or comparable measure >0.95 OR RMSEA<br><0.06 OR SRMR <0.08<br>**IRT/Rasch**:<br>No violation of unidimensionality: CFI or TLI or comparable<br>measure >0.95 OR RMSEA <0.06 OR SRMR <0.08<br>*AND*<br>no violation of local independence: residual correlations among the items after controlling for the dominant factor <<br>0.20 OR Q3's < 0.37<br>*AND*<br>no violation of monotonicity: adequate looking graphs OR item<br>scalability >0.30<br>*AND*<br>adequate model fit:<br>IRT: $\chi 2$ >0.01<br>Rasch: infit and outfit mean squares ≥ 0.5 and ≤ 1.5 OR Zstandardized<br>values > -2 and <2 |
| | ? | CTT: Not all information for '+' reported<br>IRT/Rasch: Model fit not reported |
| | - | Criteria for '+' not met |
| **Internal consistency** | +<br>?<br>– | At least low evidence for sufficient structural validity AND Cronbach's alpha(s) ≥ 0.70 for each unidimensional scale or subscale<br>Criteria for "At least low evidence for sufficient structural validity" not met<br>At least low evidence for sufficient structural validity AND Cronbach's alpha(s) < 0.70 for each unidimensional scale or subscale |
| **Reliability** | +<br>?<br>– | ICC or weighted Kappa ≥ 0.70<br>ICC or weighted Kappa not reported<br>ICC or weighted Kappa <0.70 |
| **Measurement error** | +<br>?<br>– | SDC or LoA < MIC<br>MIC not defined<br>SDC or LoA > MIC |
| **Hypothesis testing for construct validity** | +<br>?<br>- | The result is in accordance with the hypothesis<br>No hypothesis defined (by the review team)<br>The result is not in accordance with the hypothesis |

| | | |
|---|---|---|
| **Cross-cultural validity/Measurement Invariance** | + | No important differences found between group factors (such as age, gender, language) in multiple group factor analysis OR<br>no important DIF for group factors (McFadden's R2 < 0.02) |
| | ? | No multiple group factor analysis OR DIF analysis performed |
| | - | Important differences between group factors OR DIF was<br>Found |
| **Criterion validity** | + | Correlation with gold standard ≥ 0.70 OR AUC ≥ 0.70 |
| | ? | Not all information for '+' reported |
| | - | Correlation with gold standard < 0.70 OR AUC < 0.70 |
| **Responsiveness** | + | The result is in accordance with the hypothesis OR AUC ≥ 0.70 |
| | ? | No hypothesis defined (by the review team) |
| | - | The result is not in accordance with the hypothesis OR AUC <0.70 |

**Table S6.** Characteristics of oral health assessment instruments

| Tools | Purpose | Expertise of developers | Development | Administration | Scoring |
|---|---|---|---|---|---|
| **AREALD-30 [54]** | To introduce an oral health literacy instrument for Arabic speaking population and evaluate its psychometric properties. | Dental professionals and translators. | Developed by translating words from the pool of English REALD-99 words into Arabic language. | Words to be read aloud by the respondent in the interviews conducted by two bilingual investigators. | Total score ranged from 0 to 30. Each immediate correct pronouncing of the word received one mark, while 0 marks was given for pauses, hesitations, and repetitions. |
| **Brazilian-HeLD [61]** | To develop a valid and reliable tool to measure broad aspects of OHL construct for an elderly Brazilian population. | Dental researchers with translation experience and knowledge of OHL, Brazilian- Portuguese teacher and a linguistic researcher. | Developed by translating the original English HeLD scale into Brazilian Portuguese language. | Interviewer-administered questionnaire | Summary scores ranged from 0–116 (HeLD-29) and 0–56 (HeLD-14). Each item is scored using 5-point ordinal items ranging from 0 ('Unable to do') to 4 ('without any difficulty'). |
| **BOHLAT-P [62]** | To cross-culturally adapt and validate functional OHL instrument in Brazilian context, which is also directed towards paediatric dentistry. | Paediatric dentists and Professional translators/ Researchers' expertise in the field of Paediatric dentistry. | Developed by translating the English version of HKOHLAT-P, which was based on TOFHLiD items into Brazilian-Portuguese language. | Oral health knowledge was assessed by displaying pictures and asking respondents to name the pointed structures. Numeracy test had four questions groups which participants had to read and interpret information. Comprehension test involves conversation between the dentist and a parent, with blank parts to be completed according to the response options provided | Total score ranges from 0 to 49points instead of 52 as per the original instrument due to minor alterations in questionnaire, higher score indicating a higher OHL level. |

| | | | | | |
|---|---|---|---|---|---|
| **BREALD-30 [55]** | To perform cross-cultural adaption and validation of OHL tool in Brazilian population. | Panel consisting of researchers, two translators, three dentists and dental specialists with knowledge regarding health education assessment and fluent English. | Developed by translating REALD-30 into Brazilian-Portuguese language. Twenty new words were added, and some words of the original instrument were changed to maintain the ascending order of reading difficulty. | Words to be read aloud by the respondent in the interviews conducted by a trained investigator. | Total score ranges from 0 (lowest degree of literacy) to 50 (highest degree of literacy). |
| **BREALMD-20 [56]** | To perform Brazilian cross-cultural adaptation and validation of an OHL instrument focused on simultaneous recognition of dental and medical terms. | Experts in health education, bilingual health professionals, language and communication specialist. | Developed by translating the original version of REALMD-20 into Brazilian-Portuguese language. Some terms were replaced by equivalent terms and arranged in order of increasing difficulty. | Words to be read aloud by the respondent in the interviews conducted by a trained investigator. | Total score ranges from 0 to 20, each score was given to clearly pronounced words, whereas silence, hesitation, "trial and error", mispronunciation, or not attempted words received 0. |
| **HKREALD-30 [57]** | To develop and evaluate a locally relevant OHL instrument in Chinese. | Four trilingual (Cantonese/Putonghua/ English) and bi-literate (Chinese/English) researchers,<br><br>including two pediatric dentists and a dental hygienist. | Developed by translating the originalREALD-99, modelled after REALM into Chinese language. Modifications were made in the order of words. | Words to be real aloud by the participant in the interviews conducted by research assistant. | Total score ranged from 0 to 30.<br><br>Each immediate correctly pronounced word received 1 mark; pauses, hesitations and repetitions received a zero mark. |
| **IREALD-99 [58]** | To develop and validate an OHL instrument for the use in | Translators and project manager. Expertise not reported. | Developed by translating English REALD-99 based on REALM into | Words to be read aloud in the interviews conducted by two interviewers. | Total score ranged from 0 to 99.<br><br>A correct response of word is rated 1, while pauses, hesitations and |

| | | | | | |
|---|---|---|---|---|---|
| | Iranian population. | | Persian language. | | repetitions are rated 0. |
| **OHLA-B [63]** | To generate OHL instrument in Brazilian Portuguese that presents different ways of evaluating literacy. | Expert committee consisting of four dentists, Spanish and Portuguese teachers and translators. | Developed by translating the Spanish OHLA-S into Brazilian Portuguese which uses only 24 words from the vocabulary of REALD-30. | Words displayed by the interviewer to be pronounced in face-to-face interviews. | Each item was assigned 1 score when both pronunciation and association tests were correct. The item scored 0 if either of the results were incorrect. |
| **OHL-AQ-H [64]** | To culturally adapt and examine reliability and validity of a stable comprehensive OHL tool for Hindi-speaking population. | Experts in public health, the original translator, and experts in translation and development of questionnaires. | Developed by translating the original OHL-AQ into Hindi language. | Face to face interviews carried out by the primary investigator. | Total score ranged from 0 to 17, 0-9 =Low OHL, 10-11= Moderate OHL and 12-17=High OHL. |
| **OHLI-Cl [66]** | To develop and culturally adapt tool measure OHL in Spanish speaking population in South America. | Professional translators and experts in dental public health. | Developed by translating the original OHLI based on TOFHLiD into Spanish language. Minor modifications were made to maintain difficulties and understanding of questionnaire. | Consists of cloze-procedure based reading comprehension and numeracy sections. Four possible answers were given for each omitted word, among which one was correct and others either sounded similar or grammatically incorrect. | Total score ranged from 0 to 100, 0-50 weighted score for each section Each item scored 1 for correct answer or 0 for incorrect or unanswered items. The scores of reading comprehension section and numeracy sections were multiplied by 1.316 (50/38) and 2.632 (50/19) respectively. |
| **OHLI-M [65]** | To develop a functional OHL instrument for Malaysian population. | Specialist, lecturers, and doctorate student's expertise in dental public health, along with translators proficient in English as well as their native language. | Developed by translating the original OHLI, based on TOFHLiD into Malay language. Minor modifications were made in passage reading to fit the Malaysian context. | Reading section is self-administered where respondents have to choose a correct answer from 4 possible choices for each item and numeracy section administered by | Total score ranged from 0 to 100, 0-50 weighted score for each section. Each item scored 1 for correct answer or 0 for incorrect or unanswered items. The scores of reading comprehension section and numeracy sections were multiplied |

| | | | | | |
|---|---|---|---|---|---|
| | | | | face-to-face interviews. | by 1.316 (50/38) and 2.632 (50/19) respectively. |
| **REALD-30-for Chilean population [59]** | To report the adaptation and validation o REALD-30 for the Chilean population or the other Spanish-speaking population in South America. | A professional translator, two dentists and four experts in dental public health. | Developed by translating the original REALD-30 into Spanish language. | Each participant was asked to read aloud the provided list of words. | The total score ranged from 0 to 30. Each immediate correct pronouncing of the word received one mark, while 0 marks was given for pauses, hesitations, and repetitions. |
| **R-OHLI [67]** | To develop and examine reliability and validity of OHL instrument that followed the actual oral health related material in Belarus. | Not stated. | Developed by translating the original OHLI based on TOFHLiD into Russian language. Minor modifications were made to suit the routine dental services in Belarus. | Consists of cloze-procedure based reading comprehension and numeracy sections. Four possible answers were given for each omitted word, among which one was correct and others either sounded similar or grammatically incorrect. | Total score ranged from 0 to 100, 0-50 for each section.<br>Each item received 1 score for correct answer or 0 for incorrect or unanswered items. The scores of reading comprehension section and numeracy sections were multiplied by 1.316 (50/38) and 2.632 (50/19) respectively. |
| **RREALD-30 [60]** | To translate the REALD-30 into Romanian and test its validity and reliability in the context of urban Romanian adults. | Dentists and sociologists. | Developed by translating the original REALD-30 into Romanian language | Words to be read aloud in the interviews conducted by two researchers. | The total score ranged from 0 to 30. Each immediate correct pronouncing of the word received one point. |
| **ThREALD-30 [68]** | To create OHL tool and evaluate its reliability and validity for patients in Thailand | Experts of dentistry and linguistics. | Developed by translating the original REALD-30 into Thai language. | Words to be read aloud in the interviews conducted. | Total score ranged from 0 to 30.<br>One point was given for each correctly pronounced word |

| | | | | | |
|---|---|---|---|---|---|
| **TREALD-30 [69]** | To develop OHL tool and evaluate its psychometric properties for Turkish-speaking groups. | Six oral health professionals with expertise in public dental health, paediatric dentistry, and oral surgery, oral radiology, a physician, a biostatistician, one linguist and four translators. | Developed by translating the original REALD-30 into Turkish language. Few changes were made to increase conceptual and semantic equivalence. | Words to be read aloud participants in interviews conducted by trained interviewer. | Total score ranged from 0 to 30.<br>One point was given for each correctly pronounced word. |

**Table S7.** Assessment of quality of translation and cross-cultural adaptation of oral health literacy tools into different languages

| Oral health literacy tools | Language | Translation | Synthesis | Back Translation | Expert Committee review | Pretesting |
|---|---|---|---|---|---|---|
| AREALD-30 [54] | From English to Arabic | + | + | + | + | + |
| Brazilian-Held [61] | From English to Brazilian Portuguese | + | 0 | + | + | + |
| BOHLAT-P [62] | From English to Brazilian Portuguese | + | + | + | + | + |
| BREALD-30 [55] | From English to Brazilian Portuguese | + | + | + | + | + |
| BREALMD-20 [56] | From English to Brazilian Portuguese | + | + | + | 0 | + |
| HKREALD-30 [57] | From English to traditional Chinese | + | + | ? | + | + |
| IREALD-99 [58] | From English to Persian | + | + | + | 0 | + |
| OHLA-B [63] | From Spanish to Brazilian Portuguese | + | + | + | + | + |
| OHL-AQ-H [64] | From English to Hindi | - | 0 | - | + | + |
| OHLI-Cl [66] | From English to Spanish | + | + | + | + | + |
| OHLI-M [65] | From English to Malay | + | + | + | + | + |
| REALD-30 for Chilean population [59] | From English to Spanish | + | + | 0 | ? | 0 |
| R-OHLI [67] | From English to Russian | - | 0 | - | 0 | 0 |
| RREALD-30 [60] | From English to Romanian | + | + | - | ? | + |
| ThREALD-30 [68] | From English to Thai | + | + | ? | ? | + |

| | | | | | | |
|---|---|---|---|---|---|---|
| TREALD-30 [69] | From English to Turkish | + | + | + | + | + |

? Positive rating; ? indeterminate rating; - negative rating; 0 no information available

**Table S8.** Rating of the measurement properties per language and tool using the criteria for the good psychometric properties (Prinsen et al., 2018)

| Language | Tool | Structural Validity | Internal Consistency | Reliability | Measurement Error | Hypothesis Testing for Construct Validity | Cross-cultural Validity/ Measurement Invariance | Responsiveness |
|---|---|---|---|---|---|---|---|---|
| Arabic | AREALD-30 [54] | **-**<br>CFI=0.89<br>RMSEA=0.14 | **+** Cronbach's alpha = 0.89 | **+**<br>ICC= 0.992 | **NR** | Spearman correlation coefficients of AREALD-30 with AREALD-99=0.959, A-OHIP-14= -0.105 Self-perceived oral health status= 0.136 and Dental visiting habits= -0.142 | **NR** | **NR** |
| Chinese | HKREALD-30 [57] | **?** | **+** Cronbach's alpha=0.84 | **+**<br>ICC=0.78 | **NR** | Spearman correlation coefficients of HKREALD-30 with HKREALD-99 =0.869,<br>TOFHLiD =0.693 and reading habits: printing materials = 0.389 and digital material=0.287 | **NR** | **NR** |
| Hindi | OHL-AQ-H [64] | **NR** | + Cronbach's alpha=0.70 | +<br>ICC=0.93 | **NR** | Correlation coefficients not calculated | **NR** | **NR** |
| Malay | OHLI-M [65] | **NR** | **+** Cronbach's alpha=0.88 | **+**<br>ICC=0.86 | **NR** | Spearman's correlation with S-TOFHLA-M = 0.37 Pearson's correlation coefficient with DMFT and CPI = -0.11 and0.04 respectively. | **NR** | **NR** |
| Persian | IREALD-99 [58] | **?** | **+** Cronbach's alpha=0.98 | **+**<br>**ICC=0.97** | **NR** | Spearman's correlation with TOFHLiD spearman's =0.72 and self-perceived dental health status = 0.31. | **NR** | **NR** |
| Portuguese | Brazilian-HeLD [61] | Data 1<br>CFI=0.92,<br>SRMR=0.07,<br>RMSEA=0.09<br>Data 2 **(+)**<br>CFI=0.95,<br>SRMR=0.06,<br>RESEA=0.08 | **+**<br>Cronbach's alpha=0.94 for HeLD-29 and 0.89 for HeLD-14 | **NR** | **NR** | **NR** | **NR** | **NR** |

| | | | | | | | | |
|---|---|---|---|---|---|---|---|---|
| | BOHLAT-P [62] | + CFI= 0.934, TLI=0.931, RMSEA=0.041 | + Cronbach's alpha = 0.92 | + ICC=0.95 | **NR** | Spearman's Correlation with BREALD-30=0.704 , B-ECOHIS=-2.30 Years of schooling=0.602 Hours spent reading=0.342 and Number of teeth with cavitated caries= −.158. | **NR** | **NR** |
| | BREALD-30 [55] | **?** | +Cronbach's alpha = 0.88 | +ICC= 0.983 | **NR** | Spearman's correlation with NFLI=0.593 , educational attainment = 0.541), OHIP-14 =-0.08, monthly household income= 0.327. | **NR** | **NR** |
| | BREALMD-20 [56] | **?** | + Cronbach's alpha = 0. 789 | + ICC=0.73 | **NR** | Spearman's Correlation with BREALD-30 = 0.73 BNFLI =0.60. | **NR** | **NR** |
| | OHLA-B [63] | **NR** | **NR** | **NR** | **NR** | **NR** | **NR** | **NR** |
| Romanian | RREALD-30 [60] | + (AIC = 3443.97, BIC =3532.67, RMSEA =0). | + Cronbach's alpha = 0.88 | + **ICC= 0.90** | **NR** | Correlation coefficients not calculated | **NR** | **NR** |
| Russian | R-OHLI [67] | **NR** | + Cronbach's alpha = 0. 895 | + ICC=0.875 | **NR** | Pearson's correlation coefficients between R-OHLI and oral health knowledge test = 0.363. | **NR** | **NR** |
| Spanish | OHLI-Cl [66] | **NR** | + Cronbach's alpha =0.887 | + ICC=0.79 | **NR** | Pearsons (r) and Spearmans (p) correlation with: OHKT r = 0.673 p= 0.690, SAHLSA r= 0.560 and p=0.605, DMFT Index r = -0.329 p = -0. 321, CPI r = -0.227 p = -0.250, OHIS r = -0.209 p = -0.203, OHIP-49sp r = -0.209 p = -0.235 | **NR** | **NR** |
| | REALD-30- for Chilean population [59] | **NR** | + Cronbach's alpha = 0.876 | + ICC=0.789 | **NR** | Covergent validity Correlation with SAHLSA Pearson's r= 0.719; Spearman's rho = 0.693 Predictive validity CPI r = -0.250; rho = -0.252 | **NR** | **NR** |

| | | | | | | | | |
|---|---|---|---|---|---|---|---|---|
| Thai | ThREALD-30 [68] | **NR** | + Cronbach's alpha = 0.95 | + **ICC**=0.970 | **NR** | OHIS r = -0.138; rho = -0.141<br>DFMT r = -0.279; rho= -0.270<br>OHIP-49sp r = -0.171; rho = -0.170<br>Spearman's correlation with OHIP-14 =−0.688, DMFT = −0.283, OHIS=−0.432, and CAL = −0.470. | **NR** | **NR** |
| Turkish | TREALD-30 [69] | **-**<br>CFI=0.89, TLI=0.89, and RMSEA=0.052. | +<br>Cronbach's alpha = 0.91 | +<br>ICC= 0.99 | **NR** | Spearman's correlation with REALM=0.73, OHIP-14=0.28, self-rated oral health =0.34, reading ability of hospital materials =0.69, perceived confidence in completing Medical forms = 0.59. | **NR** | **NR** |

1 "+" = sufficient," −" = insufficient, "?" = indeterminate

AIC= Akaike information criterion, BIC= Bayesian Information Criteria, CFI = comparative fit index, ICC = Intraclass correlation coefficient, RMSEA: Root Mean Square Error of Approximation, SRMR: Standardized Root Mean Residuals, TLI = Tucker-Lewis Index

**Table S9.** Methodological Quality Assessment of Studies on Psychometric Properties of the Included Tools using COSMIN risk of bias checklist

| **Language** | **Tool and study** | **Structural Validity** | **Internal Consistency** | **Cross-cultural Validity/ Measurement Invariance** | **Reliability** | **Measurement Error** | **Construct Validity** | **Responsiveness** |
|---|---|---|---|---|---|---|---|---|
| Arabic | AREALD-30 [54] | Adequate | Very good | NR | Inadequate | NR | Very good | NR |
| Chinese | HKREALD-30 [57] | Adequate | Very good | NR | Inadequate | NR | Very good | NR |

| | | | | | | | | |
|---|---|---|---|---|---|---|---|---|
| Hindi | OHL-AQ-H [64] | NR | Very good | NR | Adequate | NR | Inadequate | NR |
| Malay | OHLI-M [65] | NR | Very good | NR | Doubtful | NR | Adequate | NR |
| Persian | IREALD-30 [58] | Inadequate | Very good | NR | Doubtful | NR | Adequate | NR |
| Portuguese | Brazilian-HeLD [61] | Very good | Very good | NR | NR | NR | NR | NR |
| | BOHLAT-P [62] | Inadequate | Very good | NR | Adequate | NR | Very good | NR |
| | BREALD-30 [55] | Adequate | Very good | NR | Inadequate | NR | Adequate | NR |
| | BREALMD-20 [56] | Adequate | Very good | NR | Inadequate | NR | Very good | NR |
| | OHLA-B [63] | NR | NR | NR | NR | NR | NR | NR |
| Romanian | RREALD-30 [60] | Very good | Very good | NR | Inadequate | NR | Inadequate | NR |
| Russian | R-OHLI [67] | NR | Very good | NR | Inadequate | NR | Inadequate | NR |
| Spanish | OHLI-Cl [66] | NR | Doubtful | NR | Doubtful | NR | Adequate | NR |
| | REALD-30 for Chilean population [59] | NR | Doubtful | NR | Doubtful | NR | Adequate | NR |

| | | | | | | | | |
|---|---|---|---|---|---|---|---|---|
| Thai | ThREALD-30 [68] | NR | Doubtful | NR | Doubtful | NR | Inadequate | NR |
| Turkish | TREALD-30 [69] | Inadequate | Very good | NR | Adequate | NR | Adequate | NR |
